# Hashimoto’s Thyroiditis Minimizes Lymph Node Metastasis in *BRAF* Mutant Papillary Thyroid Carcinomas

**DOI:** 10.3390/biomedicines10082051

**Published:** 2022-08-22

**Authors:** Peter P. Issa, Mahmoud Omar, Yusef Buti, Chad P. Issa, Bert Chabot, Christopher J. Carnabatu, Ruhul Munshi, Mohammad Hussein, Mohamed Aboueisha, Mohamed Shama, Ralph L. Corsetti, Eman Toraih, Emad Kandil

**Affiliations:** 1 School of Medicine, Louisiana State University Health Sciences Center, New Orleans, LA 70112, USA; 2 Department of Surgery, School of Medicine, Tulane University, New Orleans, LA 70112, USA; 3 Genetics Unit, Department of Histology and Cell Biology, Faculty of Medicine, Suez Canal University, Ismailia 41522, Egypt

**Keywords:** thyroid cancer, Hashimoto’s thyroiditis, lymph node metastasis, risk factor, protective factor

## Abstract

Hashimoto’s thyroiditis (HT) (autoimmune thyroiditis) is a clinicopathological entity associated with chronic lymphocytic infiltration resulting in hypothyroidism. HT is a double-edged sword that increases the risk of papillary thyroid cancer (PTC), yet it serves as a protective factor for PTC progression. *BRAF* mutation in PTCs is associated with rapid cell growth, aggressive tumor characteristics, and higher mortality rates. Here, we aimed to analyze the influence of HT in patients with PTCs and its effect on lymph node metastasis (LNM) in BRAF mutant tumors. Adults diagnosed with PTC between 2008 and January 2021 were retrospectively included. A total of 427 patients, 128 of whom had underlying HT, were included. The HT group had significantly higher rates of microcarcinoma (49.2% vs. 37.5%, *p* = 0.025) and less lateral LNM (8.6% vs. 17.1%, *p* = 0.024). Interestingly, *BRAF*-mutated PTCs were found to have significantly less overall LNM (20.9% vs. 51%, *p* = 0.001), central LNM (25.6% vs. 45.1%, *p* = 0.040) and lateral LNM (9.3% vs. 29.4%, *p* = 0.010) in patients with HT when compared to those without underlying HT. HT was found to be an independent protective predictor of overall and lateral LNM. Altogether, HT was able to neutralize the effect of *BRAF* mutation and was determined to be an independent protective factor against LNM. Specifically, our work may influence treatment-aggressiveness decision making for endocrinologists, oncologists and surgeons alike.

## 1. Introduction

Papillary thyroid carcinoma (PTC) makes up 90% of all thyroid cancers, making it the most common endocrine malignancy [1]. As of 2013, thyroid cancer was the fastest growing cancer in the United States [2]. With continued increased imaging studies and genetic testing, allowing for increased diagnostic scrutiny, the prevalence of thyroid cancer, including PTC, is expected to continue to increase [3,4]. Though PTC patients generally have an excellent prognosis, the rate of recurrence can be as high as 30% [5], and consequently factors which can predict PTC aggressiveness and recurrence are important. A common risk factor is *BRAF^V600E^* mutation, which is prevalent in up to 51% of PTCs [6]. Mutation in the BRAF oncogene *BRAF^V600E^* is widely associated with advanced cancer, lymph node metastasis (LNM), and decreased patient 10-year survival rate [7,8,9].

Hashimoto’s thyroiditis (HT) (also known as autoimmune thyroiditis) is the most common thyroid-related autoimmune disorder characterized pathophysiologically by lymphocytic infiltration and a hypoactive thyroid gland [10]. HT is the leading cause of hypothyroidism [11]. The co-occurrence of HT and PTC was first described in the 1950′s and, considering their elevated concomitant prevalence (as high as 58%), are thought to influence one another [12]. Due to the nature of HT, however, as a disease that infiltrates, destroys and replaces thyroid cells, the notion of less-aggressive-PTCs has been suggested [12,13,14].

A recent work looking at HT and its ability to serve as a protective marker found that the disease decreased primary PTC size and lymph node involvement [15]. Considering this, along with the respectable prevalence of concomitant HT and PTC, we sought to further investigate the protective effectiveness of HT in PTC patients. Specifically, we aimed to analyze the influence of HT in patients with PTCs and its effect on LNM in *BRAF* mutant tumors.

## 2. Methods

### 2.1. Study Design & Recruited Cohort

Following institutional review board approval at Tulane University, this retrospective study was conducted. Patients diagnosed with PTC and undergoing thyroid surgery between 2008 and 2021 were included. Surgical operations included hemithyroidectomy, total thyroidectomy, total thyroidectomy with central lymph node dissection, and total thyroidectomy with both central and lateral lymph node dissection. Patient demographics, tumor cytopathological data, operative details, and pathological parameters of interest, including tumor-nodal-metastasis (T-N-M) staging, extrathyroidal extension, and disease recurrence, were collected.

### 2.2. Determination of BRAF Mutation and Hashimoto’s Thyroiditis Status

All patients were evaluated for *BRAF* mutation and HT status. Evaluation for genetic mutation status was conducted either preoperatively via fine-needle aspiration (FNA) sampling and/or core needle biopsy (CNB) or postoperatively via tumor specimen analysis. Preoperative biopsy cytology was analyzed using Interpace ThyGenX/ThyraMIR (Interpace Biosciences, Parsippany, NJ, USA) or Afirma Thyroid FNA Analysis (including both GEC and GSC; Veracyte Inc., San Francisco, CA, USA). The majority of nodules were evaluated twice preoperatively, although a small minority were analyzed only once. Surgical specimen *BRAF^V600E^* mutational analysis was performed by the University of Pittsburgh Medical Center and analyzed by real-time polymerase chain reaction (PCR). DNA extraction from formalin-fixed, paraffin-embedded frozen sections proceeded using a Qiagen EZ1 tissue kit (Qiagen, Hilden, Germany) and was subject to a validated *BRAF* mutation kit (EntroGen, Woodland Hills, CA, USA) with a sensitivity of 1–5% in a background of wild-type genomic DNA.

The diagnosis of HT was made in the following scenarios: (A) either overt or subclinical hypothyroidism with sonographically moderate or prominent heterogenous thyroid gland as well as anti-thyroglobulin (TgAb) >40 U/mL, and/or anti-thyroid peroxidase antibody (TPOAb) > 50 U/mL, (B) Histopathological diagnosis defined by existence of diffuse lymphocytic infiltration with lymphoid follicles formation and the presence of reactive germinal centers among patients with hypothyroidism or euthyroid status.

### 2.3. Statistical Analysis

Descriptive statistics summarizing patient demographics, operative details, and pathological parameters of interest were sub-grouped by patient underlying HT status. Subsequently, univariate analyses sub-grouped by patient underlying HT status were conducted to determine the effect (protective or risk) of LNM incidence in wild-type *BRAF* PTCs and *BRAF* mutant PTCs. Subsequently, a multivariate analysis was conducted to determine independent predictors of LNM in *BRAF* mutant PTCs.

## 3. Results

### 3.1. Characteristics of the Study Population

The total number of patients with PTC who had undergone thyroid surgery was 427. The number of patients who did and did not have underlying HT was 128 (30.0%) and 299 (70.0%), respectively (Table 1). The number of patients below the age of 55 years did not differ between the two cohorts (*p* = 0.11). With respect to both race and sex, the number of Whites and females with HT in our study population was significantly greater than those without HT (*p* = 0.007; *p* = 0.029; respectively). These differences were expected, considering that autoimmune disease is more prevalent in both white and female populations [16]. A total of 145 patients had BRAF mutations, including 102 (34.1%) patients without underlying HT and 43 (33.6%) patients with underlying HT (*p* = 0.92).

With respect to the pathological data, significant differences were seen between the two groups. Patients with HT were more likely to have tumors diagnosed as microPTCs (HT: 49.2%; no HT: 37.5%; *p* = 0.025). In addition, HT patients were less likely to have any lymph node involvement (HT: 18%; no HT: 27.8%; *p* = 0.037), lateral lymph node involvement (HT: 8.6%; no HT: 17.1%; *p* = 0.024), and extranodal extension (HT: 4.7%; no HT: 10.7%; *p* = 0.046). Extrathyroidal extension (*p* = 0.09), multifocal disease (*p* = 0.16) and metastasis (*p* = 0.07) tended to occur less frequently in the cohort of HT patients.

### 3.2. Association of HT with Lymph Node Metastasis

**Figure 1:**
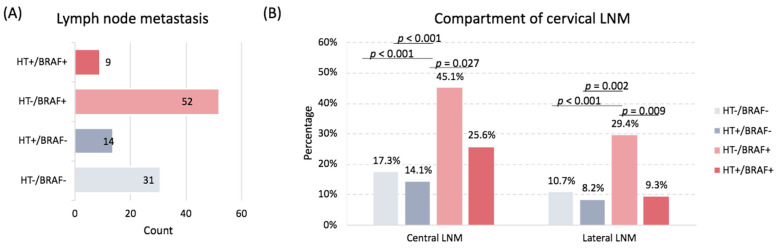
Frequency of lymph node metastasis (LNM) according to patient Hashimoto’s thyroiditis (HT) and *BRAF* mutation status. (**A**) Number of LNM overall. (**B**) Frequency of LNM by cervical compartment.

A total of 106 patients presented with LNM at the time of diagnosis (24.8%). 61 (57.5%) had BRAF mutation. Of those with BRAF mutation (N = 61), only 9 (14.8%) were in patients with underlying HT. Altogether, 103 (24.1%) patients and 62 (14.5%) patients had central and lateral cervical compartment infiltration, respectively. The location and frequency of cervical LNM stratified by the presence and/or absence of HT, as well as the presence and/or absence of *BRAF* mutation, are depicted in Figure 1. The frequency of LNM was highest in patients harboring *BRAF* mutant PTCs but without HT (49.1%). When LNM was stratified by compartment, HT continued to display a protective effect. Patients with *BRAF* mutant PTCs without underlying HT had higher rates of central (45.1% vs. 25.6%, *p* = 0.027) and lateral LNM (29.4% vs. 9.3%, *p* = 0.009) when compared to patients with HT. Similarly, patients with wild-type *BRAF* PTCs without underlying HT had higher rates of central (17.3% vs. 14.1%; *p* < 0.001) and lateral LNM (10.7% vs. 8.2%; *p* < 0.001) when compared to patients with HT.

The univariate risk analysis for the incidence of LNM at the time of presentation is depicted in Table 2. In general, HT patients were less likely to present with LNM (Odds ratio (OR) = 0.57, 95%CI = 0.34–0.95, *p* = 0.033). When analyzing only wild-type *BRAF* PTCs, however, HT elected neither a protective nor adversative effect in the risk of LNM incidence (OR = 1.06, 95%CI = 0.53–2.10, *p* = 0.87). When considering *BRAF* mutant PTCs, HT was associated with a 75% reduced risk of lymph node infiltration when compared to patients without underlying HT (OR = 0.25, 95%CI = 0.11–0.58, *p* = 0.001). Specifically, HT patients harboring *BRAF* mutant PTCs were less likely to develop central (OR = 0.42, 95%CI = 0.19–0.92, *p* = 0.030) and lateral LNM (OR = 0.24, 95%CI = 0.08–0.75, *p* = 0.014) compared to the patient cohort without underlying HT.

### 3.3. Independent Predictors of Lymph Node Metastasis in BRAF-Mutated Tumors

**Figure 2:**
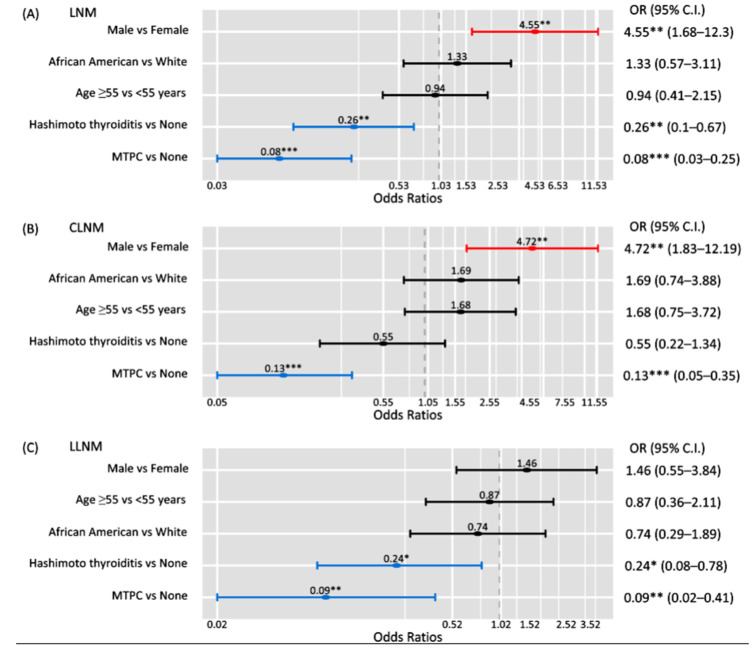
Multivariate logistic regression analysis for determining independent predictors of lymph node metastasis (LNM) in patients with *BRAF* mutant PTCs. (**A**) LNM overall. (**B**) Central LNM (CLNM). (**C**) Lateral LNM (LLNM). * indicated *p* < 0.05; ** indicated *p* < 0.01; *** indicates *p* < 0.001.

Three predictors of LNM were determined, one of which was a risk factor and two of which were protective factors. The independent predictors of LNM are depicted in Figure 2. Patients with *BRAF* mutant PTCs were more than four times as likely to be male (OR = 4.55, 95%CI = 1.68–12.3; *p* < 0.01). Interestingly, microPTCs or the presence of underlying HT had 91% (OR = 0.09, 95%CI = 0.02–0.41; *p* < 0.001) and 76% (OR = 0.24, 95%CI = 0.08–0.78; *p* < 0.01) decreased odds of developing LNM in patients with *BRAF* mutant PTC. These findings were carried over when analyzing LNM by cervical compartment. Specifically, male sex (OR = 4.72, 95%CI = 1.83–12.19; *p* < 0.01) and microPTC (OR = 0.13, 95%CI = 0.05–0.35; *p* < 0.001) continued to serve as risk and protective factors, respectively, for central LNM. With regards to lateral LNM, both HT (OR = 0.24, 95%CI = 0.08–0.78; *p* < 0.05) and microPTC (OR = 0.09, 95%CI = 0.02–0.41; *p* < 0.01) continued to be independent predictors of protection.

## 4. Discussion

PTC comprises the vast majority (90%) of thyroid malignancies and is the fastest growing cancer in the United States [2]. In *BRAF* mutant PTCs, patient prognosis is significantly worse, leading to decreased patient survival rates as well as increased LNM, increased extrathyroidal extension, and more advanced cancer stage [7,8]. While several studies have demonstrated the risk-reducing effect of HT in PTCs, this work looked to put into perspective its protective ability via its potential to mitigate *BRAF*-mutated PTC risk. Overall, we found HT to be an independent protective factor in PTC, able to neutralize the adverse consequences associated with the *BRAF* mutation.

Several works have described the co-occurrence of PTC and HT, yet there remains debate with respect to both the pathophysiological mechanism of action of their relation and the significance of one on the other. One hypothesis suggests that diffuse lymphocytic infiltration of the thyroid gland prior to tumor formation results in inflammation and dysregulation of thyroid follicular cells, thereby promoting a trophic tumorigenic effect [17,18]. In a similar sense, some studies suggest that elevated TSH levels secondary to HT-induced hypothyroidism promote follicular epithelial proliferation [19,20,21]. On the other hand, it could be the case that malignancy induces and/or triggers an immunologic response, thereby bringing about HT [22,23,24]. With respect to their concomitant prevalence, however, the literature suggests a clear direct correlation between the two [15,21,24] as well as an overall protective effect [15,25,26,27]. When comparing our HT patient cohort to those without underlying HT, we found significantly reduced incidence rates of extranodal extension, overall LNM, and lateral LNM. A 2016 meta-analysis of 2,334 cases found HT to be a significant protective factor for central LNM [28]. Similarly, our study suggests that HT patients are almost half as likely to have LNM. Notably, lateral LNM places a patient at considerably more risk of distant metastasis than central LNM, suggesting a respectable improvement in patient prognosis.

While both the literature and current work suggest that HT mitigates PTC aggressiveness, there is limited work analyzing whether it can meaningfully ameliorate the effect of known PTC recurrence risk factors, such as *BRAF* mutation. There is an abundance of literature that associates *BRAF* mutation with an increased risk of both LNM and malignancy recurrence [29,30]. A 2012 meta-analysis associated *BRAF* mutation with more malignant cancers (i.e., advanced cancer diagnosis, LNM and extrathyroidal extension), accompanied by a two-fold increased risk of recurrence or persistent disease [7]. One work that analyzed the protective effect of HT in *BRAF*-mutated PTCs reported that patients had significantly less extracapsular extension (57.6% vs. 29.6%, *p* = 0.001) and smaller tumor sizes (*p* = 0.028), but similar rates of LNM (35.9% vs. 31.5%, *p* = 0.509) [31]. In line with HT serving as a protective factor, but in contrast to the latter findings of Marotta et al., we found that HT reduced the risk of overall LNM by 75% in patients with *BRAF* mutant PTCs. Specifically, we found that HT patients harboring *BRAF* mutant PTCs were 58% and 75% less likely to develop central and lateral LNM, respectively, when compared to patients without underlying HT. This suggests that HT has the potential to mitigate PTC aggressiveness and effectively neutralize the harmful effects of *BRAF* mutation. In univariate analysis, however, HT had a neutral effect on wild-type *BRAF* PTC LNM. Therefore, the prognostic value of HT and its protective ability are limited to *BRAF* mutated PTCs, which comprise between 45% and 51% of all PTCs [6,32].

A positive lymph node count is a risk factor for PTC recurrence [33], emphasizing the importance of determining predictors of LNM. Elucidating this association, as well as establishing clinically relevant predictors, could assist surgeons in patient risk-stratification and influence the extent of surgical resection. Predictors of LNM in PTC are well known, including larger tumor size, extracapsular invasion, and *BRAF* mutation [28,34]. On the contrary, little is known about the independent predictors of LNM in *BRAF*-mutated PTCs. Our work could potentially be the first to determine the predictive factors of LNM in patients presenting with *BRAF* mutant PTCs. We found that male sex increased the odds of LNM by more than four-fold. microPTC and HT were both found to be protective factors, reducing the odds of LNM by 88% and 74%, respectively. Furthermore, microPTC and HT continued to minimize the risk of lateral LNM, with odds reduced by 91% and 76%, respectively. Altogether, HT appears to play a considerable role in ameliorating PTC aggressiveness and improving patient prognosis. Moving forward, surgeons can recognize HT as a protective factor in thyroid cancer and utilize it when considering patient risk.

Although the *BRAF* mutation itself is associated with disease progression and worse patient prognosis, its incidence is also correlated with programmed death (PD) L1 and PD-1 expression [35,36]). PD-L1 and PD-1 are prominent cell cycle regulators mediating immunosuppression [37] which are associated with LNM in patients with PTCs [38] and have been suggested as potential prognostic biomarkers [36,39,40]. Since *BRAF*-mutated PTCs also have higher incidences of radioiodine refraction [41,42], targeted immunotherapies against PD-L1/PD-1 may be a potential avenue for investigation.

The current American Thyroid Association (ATA) guidelines recommend tumor *BRAF* status to assist in thyroid cancer stratification, with its presence placing a patient at greater risk of more aggressive disease [43]. Consequently, patients harboring *BRAF* mutated PTCs are likely to receive more aggressive treatment and are therefore at greater risk of nerve injury and postoperative complication [44]. Although our study is limited in its retrospective nature, its long-term follow-up, adequate sample size, and racially diverse sample population adequately suggest that HT was able to effectively neutralize the adversative effect of *BRAF* mutation, potentially restoring patients with HT to their non-*BRAF* mutation status. Furthermore, it could be the case that patients with HT and *BRAF* mutated microPTC are at reduced levels of risk, which may allow them candidacy for non-surgical management (active surveillance).

## 5. Conclusions

Overall, our work demonstrated that HT decreased LNM in *BRAF*-mutated PTCs and served as an independent predictor of reduced LNM. When the fields of thyroidology and oncology overlap, we suggest that HT be viewed as a protective marker that can improve patient prognosis. Specifically, our work may influence treatment-aggressiveness decision making for endocrinologists, oncologists and surgeons alike.

## Figures and Tables

**Table 1:** Baseline characteristics of thyroid cancer patients who underwent thyroid surgery.

Characteristics	Levels	Total	No Hashimoto Thyroiditis	Hashimoto Thyroiditis	*p*-Value
**Number**		427	299	128	
**Demographic data**					
Age	<55 years	248 (58.1)	166 (55.5)	82 (64.1)	0.11
	≥55 years	179 (41.9)	133 (44.5)	46 (35.9)	
Sex	Female	334 (78.2)	225 (75.3)	109 (85.2)	**0.029**
	Male	93 (21.8)	74 (24.7)	19 (14.8)	
Race	White	283 (66.3)	186 (62.2)	97 (75.8)	**0.007**
	African American	144 (33.7)	113 (37.8)	31 (24.2)	
**Pathological data**					
microPTC	T1a	175 (41)	112 (37.5)	63 (49.2)	**0.025**
T stage	T1	309 (72.4)	214 (71.6)	95 (74.2)	0.11
	T2	54 (12.6)	34 (11.4)	20 (15.6)	
	T3	56 (13.1)	43 (14.4)	13 (10.2)	
	T4	8 (1.9)	8 (2.7)	0 (0)	
N stage	N0	321 (75.2)	216 (72.2)	105 (82)	**0.037**
	N1	106 (24.8)	83 (27.8)	23 (18)	
Compartment	Central LNM	103 (24.1)	80 (26.8)	23 (18)	0.06
	Lateral LNM	62 (14.5)	51 (17.1)	11 (8.6)	**0.024**
M stage	M0	413 (96.7)	286 (95.7)	127 (99.2)	0.07
	M1	14 (3.3)	13 (4.3)	1 (0.8)	
Focality	Unifocal	250 (58.5)	182 (60.9)	68 (53.1)	0.16
	Multifocal	177 (41.5)	117 (39.1)	60 (46.9)	
Laterality	Unilateral	312 (73.1)	224 (74.9)	88 (68.8)	0.19
	Bilateral	115 (26.9)	75 (25.1)	40 (31.3)	
Extrathyroidal extension	Positive	51 (11.9)	41 (13.7)	10 (7.8)	0.09
Angioinvasion	Positive	30 (7)	24 (8)	6 (4.7)	0.22
Perineural invasion	Positive	5 (1.2)	3 (1)	2 (1.6)	0.62
Capsular invasion	Positive	94 (22)	66 (22.1)	28 (21.9)	0.96
Extranodal extension	Positive	38 (8.9)	32 (10.7)	6 (4.7)	**0.046**

Data is presented as number (percentage) or median and interquartile range (IQR). Two-sided Chi-square and Mann–Whitney U tests were used. *p*-values in bold are those which were significant.

**Table 2:** Univariate risk analysis for developing LNM at the time of presentation.

Characteristics		Total	No Hashimoto’s Thyroiditis	Hashimoto’s Thyroiditis	*p*-Value	OR (95%CI)	*p*-Value
**BRAF Wild Type**							
Overall LNM	Negative	237 (84)	166 (84.3)	71 (83.5)	0.86	*Reference*	
	Positive	45 (16)	31 (15.7)	14 (16.5)		1.06 (0.53–2.1)	0.87
Central LNM	Negative	236 (83.7)	163 (82.7)	73 (85.9)	0.60	*Reference*	
	Positive	46 (16.3)	34 (17.3)	12 (14.1)		0.79 (0.39–1.61)	0.51
Lateral LNM	Negative	254 (90.1)	176 (89.3)	78 (91.8)	0.66	*Reference*	
	Positive	28 (9.9)	21 (10.7)	7 (8.2)		0.75 (0.31–1.84)	0.53
BRAF mutant type							
Overall LNM	Negative	84 (57.9)	50 (49)	34 (79.1)	**0.001**	*Reference*	
	Positive	61 (42.1)	52 (51)	9 (20.9)		0.25 (0.11–0.58)	**0.001**
Central LNM	Negative	88 (60.7)	56 (54.9)	32 (74.4)	**0.040**	*Reference*	
	Positive	57 (39.3)	46 (45.1)	11 (25.6)		0.42 (0.19–0.92)	**0.030**
Lateral LNM	Negative	111 (76.6)	72 (70.6)	39 (90.7)	**0.010**	*Reference*	
	Positive	34 (23.4)	30 (29.4)	4 (9.3)		0.25 (0.08–0.75)	**0.014**

Data is presented as count (percentage). Two-sided Chi-square tests were performed for the comparison of frequency. Binary logistic regression analysis was carried out to identify the univariate risk of LNM in the presence of HT. Odds ratios (OR) and 95% confidence intervals (CI) were estimated. *p*-values in bold are those which were significant.

## Data Availability

Data are contained within the article.
